# Revealing the Ion Regulation Effect of Zwitterionic All‐Solid‐State Electrolytes in Lithium Metal Batteries

**DOI:** 10.1002/advs.76224

**Published:** 2026-06-22

**Authors:** Wentao Xie, Yu Wu, Yierfan, Yuling Yang, Dongdong Gao, Liangbo Xu, Luoman Qin, Xingyu Lu, Yinjuan Chen, Changle Mu, Danyu Gu, Chunlei Wei, Yi He, Gang Cheng

**Affiliations:** ^1^ School of Materials Science and Engineering Zhejiang University Hangzhou China; ^2^ School of Engineering Westlake University Hangzhou China; ^3^ School of Science Westlake University Hangzhou China; ^4^ School of Life Sciences Westlake University Hangzhou China; ^5^ Zhejiang Key Laboratory of Precise Synthesis of Functional Molecules Instrumentation and Service Center for Molecular Sciences Westlake University Hangzhou China; ^6^ Instrumentation and Service Center for Physical Sciences Westlake University Hangzhou China; ^7^ College of Chemical and Biological Engineering Zhejiang University Hangzhou China; ^8^ Institute of Zhejiang University‐Quzhou Quzhou China; ^9^ Center for Biobased Materials Muyuan Laboratory Zhengzhou China

**Keywords:** all‐solid‐state, lithium batteries, polyelectrolytes, polymer, zwitterion

## Abstract

Uniform lithium deposition is crucial for the development of lithium metal batteries, but uncontrolled lithium dendrite growth remains a major challenge. While zwitterionic materials show promise in modulating metal‐ion deposition, their underlying mechanisms remain unclear. Here, we synthesize four all‐solid‐state electrolytes with different functional groups, including a tertiary amine‐based zwitterionic polymer electrolyte (ZPE), to probe the distinct functions of its cationic and anionic sites. The “anti‐polyelectrolyte effect” is identified as a key factor governing the unique behavior of ZPE. Time‐of‐flight secondary ion mass spectrometry (TOF‐SIMS) reveals a zwitterion‐mediated, salt‐induced phase separation process that generates “ion‐enrichment pathway”‐like domains in ZPE. This heterogeneous structure gives ZPE a high Li^+^ transference number (t_Li_^+^ = 0.62) and a Li_2_O‐rich inner layer in solid electrolyte interphase (SEI), enabling uniform Li^+^ deposition. As a result, the assembled all‐solid‐state lithium metal batteries exhibit exceptional cycling stability, retaining 95% capacity retention after 500 cycles under bending. The mechanistic insight into ion regulation within ZPE provides guiding principles for next‐generation energy storage devices.

## Graphical abstract

This study investigates zwitterionic all‐solid‐state electrolytes, demonstrating strong carboxyl‐Li^+^ interaction that forms “ion‐enrichment pathway”‐like domains and manipulates Li^+^ deposition. Additionally, surface‐enriched zwitterions generate a dual‐layer SEI with an inner Li_2_O‐rich and outer LiF‐rich structure. Through ionic flux regulation and SEI engineering, zwitterions enable stable cycling in lithium metal batteries.

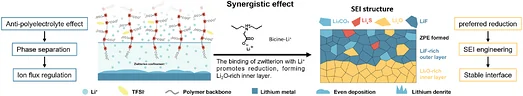

## 1 Introduction

Lithium‐ion batteries (LIBs) dominate energy storage technologies for electric vehicles, portable electronics, and grid applications. However, their energy density is limited by the capacity of graphite anodes (372 mAh g^−^^1^) [1]. Lithium metal, with its exceptional theoretical capacity (3860 mAh g^−^^1^) and low electrochemical potential (−3.04 V vs. SHE), offers a highly promising anode material for next‐generation high‐energy batteries [2, 3]. Nevertheless, conventional liquid‐electrolyte lithium metal batteries (LMBs) suffer from uncontrolled lithium dendrite growth and unstable SEIs, which severely compromise cycling stability and safety [4, 5, 6]. Replacing flammable liquid electrolytes with solid‐state electrolytes is therefore considered a critical step toward safer batteries. Among solid‐state electrolytes, solid‐state polymer electrolytes (SPEs) offer advantages in electrode interfacial compatibility, mechanical flexibility, and ability to accommodate volume changes during cycling [7].

Early studies on SPE primarily focus on poly(ethylene oxide) (PEO), polyacrylonitrile (PAN), poly(vinylidene fluoride) (PVDF), and poly(methyl methacrylate) (PMMA) systems [8]. Recently, polyurethanes (PU) have attracted significant attention as SPEs owing to their outstanding mechanical properties, desired interfacial compatibility, electrochemical stability, and ease of processing [9, 10]. ZPEs have also emerged as promising alternatives. Zwitterionic polymers contain equimolar anionic and cationic groups uniformly distributed along their polymer chains [11, 12]. Several studies unveiled the high ionic conductivity of ZPE in various organic and inorganic solvents [13, 14]. Their unique structure has inspired their application in various battery components, including additives [15, 16, 17], separators [18, 19, 20], binders [21, 22, 23], and gel/solid electrolytes [24, 25, 26]. The high dielectric constant of zwitterionic materials enhances lithium salt dissociation, thereby improving ionic conductivity and Li^+^ transference number (t_Li_^+^) [27, 28, 29]. Compared to other strategies for stabilizing lithium metal anodes, such as SEI‐forming additives, composite electrolytes, and high‐concentration electrolytes, zwitterionic polymers offer a distinct advantage. The tethered coexistence of cationic and anionic moieties on the same repeat unit enables simultaneous regulation of cation and anion transport through cooperative electrostatic interactions that promote uniform Li^+^ deposition and suppress dendrite growth.

Despite these advantages, the mechanistic understanding of how zwitterions regulate ion transport and deposition within all‐solid‐state lithium metal batteries (ASSLMBs) remains unclear. Additionally, while ZPEs and anion‐matched SPEs share identical anionic moieties but display markedly different behaviors in batteries, which has not yet been fully understood. Previous studies lack direct evidence of how zwitterions influence ion transfer and distribution at electrolyte‐electrode interfaces, particularly in ASSLMBs. Furthermore, the high ion conductivity of some reported solid‐state electrolytes may be caused by residual high‐boiling‐point solvents (e.g., DMF [30, 31], DMSO [32, 33], or ACN [34, 35]) to artificially inflate conductivity, which may not reflect their intrinsic ion‐transport mechanisms.

This study aims to elucidate a crucial ion regulation mechanism of zwitterionic polyurethane material by systematically isolating the contributions of its cationic and anionic moieties (control groups). The SPEs were prepared using methanol as the solvent due to its low boiling point. A thermogravimetric analysis/infrared spectroscopy (TGA‐IR) test was then conducted to verify the absence of any residual liquid components and eliminate interference of organic solvents. The salt‐induced phase separation phenomenon on the ZPE surface generates distinct interface behavior. Combined correlated scanning electron microscopy‐Raman (SEM‐Raman) spectra and Time‐of‐flight secondary ion mass spectrometry (TOF‐SIMS) analyses reveal distinct aggregate and homogeneous phases. We further examine how salt‐induced phase separation in ZPE affects the local distribution of ionic species. In addition, the role of the “anti‐polyelectrolyte effect” in ZPE is systematically contrasted with the “polyelectrolyte effect” observed in MPAE, to establish a unified principle underlying their divergent electrochemical behaviors. The SEI formed in lithium metal batteries is also analyzed by x‐ray photoelectron spectroscopy (XPS), Density Functional Theory (DFT), and TOF‐SIMS to correlate the heterogeneous ZPE surface with the composition of the resulting SEI. This work provides both direct visualization and mechanistic insight into zwitterion‐mediated cation/anion regulation at the electrolyte interface on a spatial scale, offering a fundamental insight for designing high‐performance ASSLMBs.

## 2 Results and Discussion

### 2.1 Structure of SPEs

**FIGURE 1:**
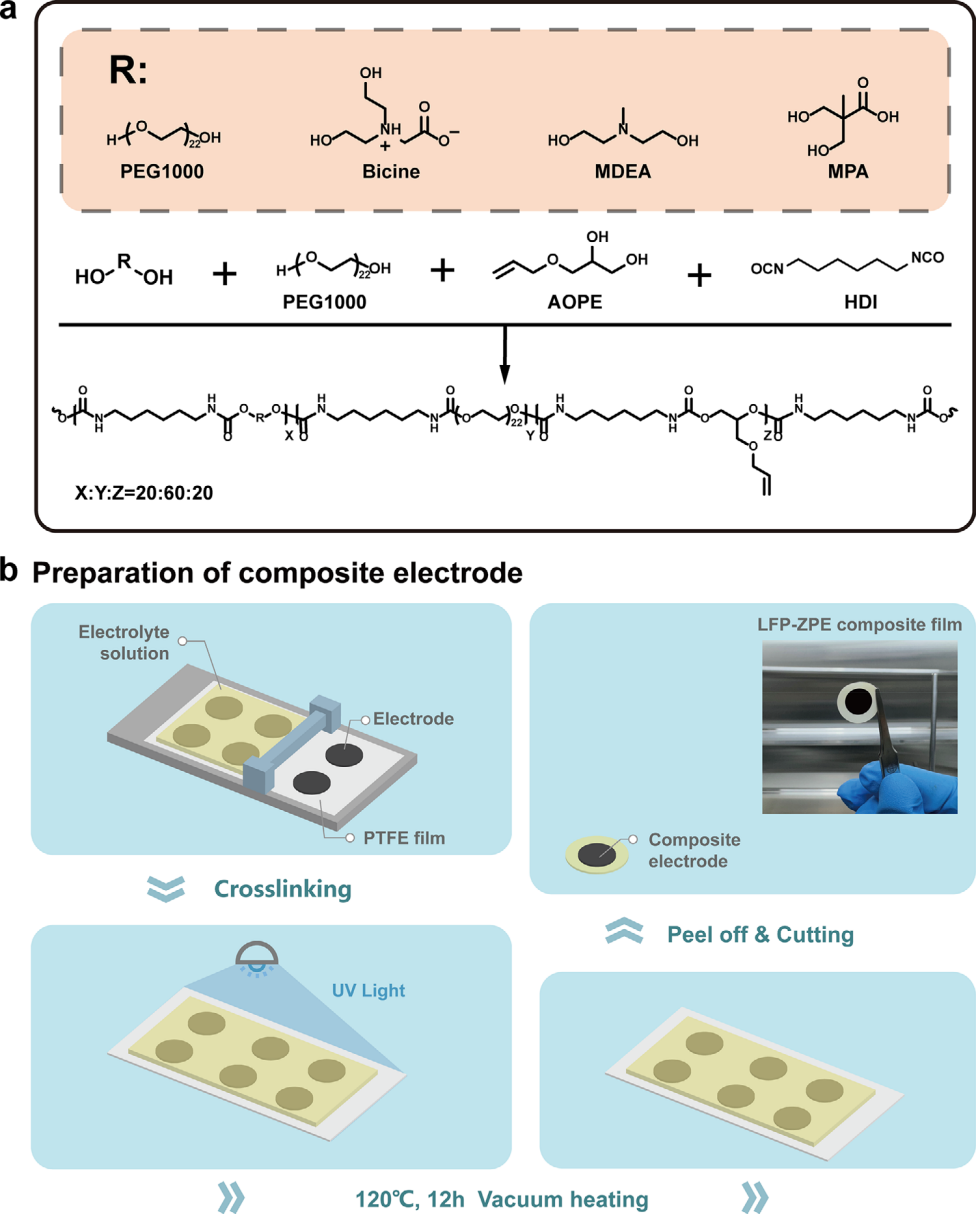
The schematic illustration of the materials synthesis and composite electrode preparation. a, Chemical structures of the PU synthesized. b, The schematic illustration of SPEs films and composite electrode preparation.

To elucidate the mechanism of zwitterions in regulating ion transport, four distinct PU electrolytes were synthesized by incorporating specific functional diols (Figure 1a). Four diols were selected based on their distinct structures and lithium‐ion conducting capabilities: PEG1000, incorporating commonly used ether groups in SPEs; bicine as a zwitterion component; N‐methyldiethanolamine (MDEA) representing the tertiary amine (cationic moiety) of bicine; 2,2‐Bis(hydroxymethyl)propionic acid (MPA) representing the carboxyl group (anionic moiety) of bicine. The resulting salt‐free PUs were designated PEGU, ZPU, MDEAU, and MPAU, respectively.

The mechanical properties of PUs were enhanced via chemical crosslinking; diols containing allyloxy groups (AOPE) were copolymerized into all PUs. Subsequent thiol‐ene click chemistry, using 2,2′‐(ethylenedioxy)diethanethiol (EDT) as a crosslinker and 2,2‐dimethoxy‐2‐phenylacetophenone (DMPA) as the photoinitiator under UV light irradiation. Following PUs synthesis and purification, lithium bis(trifluoromethanesulfonyl)imide (LiTFSI) (35 wt% relative to the total SPE mass), EDT, and DMPA were dissolved in methanol solutions of PUs together, forming the transparent precursor solution of SPEs. The LiTFSI‐containing electrolytes derived from each type of PU were correspondingly denoted as PEGE, ZPE, MDEAE, and MPAE.

To achieve good electrode‐electrolyte contact, an innovative film preparation method was developed. The precursor methanol solutions were doctor‐bladed directly onto electrode surfaces to ensure complete wetting, followed by UV‐crosslinking (Figure 1b). After crosslinking, residual solvent was removed by vacuum drying at 120°C for 12 h to obtain the final SPEs and composite electrodes.

### 2.2 Materials Structure and Mechanical Characterization

**FIGURE 2:**
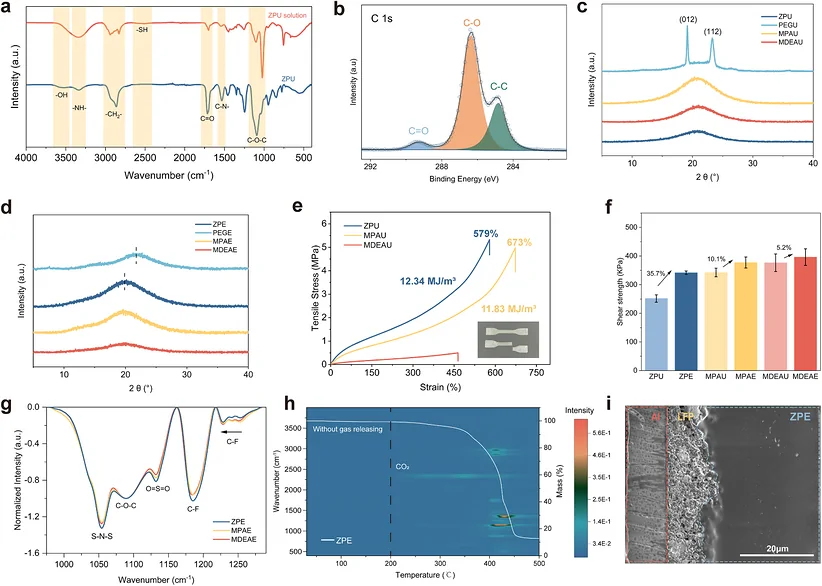
Structure characterization of the materials. (a) ATR‐FTIR spectra of the ZPU in methanol solution and ZPU film after crosslinking and drying. (b) XPS signal of ZPU. (c, d) X‐ray diffraction (XRD) patterns of the PUs (c), and the structure variation after adding LiTFSI (d). (e) Tensile test of the PUs. (f) Shear strength of the PUs and SPEs. (g) FTIR spectra of SPEs. (h) TGA curve of ZPE and the FTIR spectra of the releasing gases at different temperatures. (i) The SEM image of the composite electrode.

Precursor solution of SPEs underwent UV‐initiated thiol‐ene click crosslinking between allyloxy groups and thiols of EDT crosslinkers, converting the liquid precursor into gels (Figure S1). Attenuated total reflectance Fourier transform infrared (ATR‐FTIR) spectroscopy confirmed the completion of the crosslinking reaction and elucidated key signature groups of the products (Figure 2a and Figure S2). The thiol peak (2517 cm^−^^1^) in the precursor solution disappeared after crosslinking, confirming complete reaction and without EDT residue. Additionally, the key peaks for ZPU included urethane C═O (1715 cm^−^^1^), ─NH (3337 cm^−^^1^), PEG C‐O‐C (1092 cm^−^^1^), and hydrogen‐bonded ‐OH (3523 cm^−^^1^). XPS further confirmed the chemical structure (Figure 2b and Figure S3): C 1s spectra revealed C─C (284.8 eV), C─O─C (286.4 eV), and C═O (289.3 eV) bonds; S 2p spectra confirmed C─S bonds (163.1 eV) formed during crosslinking.

For PEO‐based SPEs, ion hopping in amorphous phases, whereas PEO crystallization severely limits ion migration in the electrolytes. The crystallinity of PUs and SPEs was characterized by XRD (Figure 2c,d). While PEGU exhibited prominent PEG crystallization peaks at 19.2° and 23.3°, copolymerization with other diols significantly disrupted chain ordering and reduced crystallinity across all PUs. Crucially, ZPU displayed the strongest suppression of PEG crystallization, in contrast to MPAU, which also contains carboxyl groups but showed the weakest suppression effect. It can be caused by the stronger inter‐ and intra‐molecular interactions of zwitterionic bicine in ZPU. The addition of LiTFSI further modulated the polymer structures, and the XRD patterns of ZPE and MPAE converged. This convergence can be explained by the “anti‐polyelectrolyte effect” [36, 37] in the zwitterionic polymer. In ZPU, strong intra‐ and interchain electrostatic attractions in the absence of salt promote chain aggregation and hinder ordering. The addition of salt screens these attractions, leading to chain relaxation and enhanced structural uniformity. Conversely, MPAU exhibits the “polyelectrolyte effect”, where the repulsion between charged chains in the salt‐free state is mitigated upon salt addition, resulting in structural convergence with ZPE. MDEAE and PEGE exhibited comparably low crystallinity, attributed to weak chain interactions and the plasticizing effect of TFSI^−^ anions [38, 39, 40]. The XRD signals of MPAE and ZPE, both containing carboxyl groups, exhibit significant differences compared to those of PEGE. This finding suggests that the packing structure within MPAE and ZPE should differ from that of PEGE.

Mechanical robustness is essential for SPEs to resist lithium dendrite penetration in LMBs. The mechanical properties of PUs were studied to investigate the impact of zwitterionic functionality. (Figure 2e and Figure S4, and Table S1). The presence of carboxyl (─COO^−^) groups in ZPU and MPAU promoted extensive hydrogen bonding, markedly improving these mechanical properties compared to MDEAU. Furthermore, coulombic interactions within the zwitterionic structure of ZPU contribute to greater toughness compared to MPAU. Due to the chemically cross‐linked structure of the material, ZPU has excellent thermal stability. ZPU can maintain its mechanical integrity at elevated temperatures, which is a key factor for enhancing battery safety and cycle life (Figure S5).

SPEs with good flexibility and adhesive properties can achieve superior interfacial contact with the electrode surface, thereby lowering contact impedance and improving battery performance [41]. The interfacial adhesion was quantified using lap‐shear tests (Figure 2f and Figure S6). In the absence of LiTFSI, MDEAU exhibited the highest shear strength (376.5 kPa), attributable to its low Young's modulus that facilitates intimate contact. Remarkably, the adhesive behavior of ZPU was profoundly influenced by the zwitterionic “anti‐polyelectrolyte effect”. While all PUs showed increased adhesion with LiTFSI addition, ZPU displayed a dramatic 35.7% enhancement in shear strength, far exceeding the increasement observed for MPAU (10.1%) and MDEAU (5.2%). This phenomenon arises because the added salts screen the electrostatic interactions between zwitterions [36, 37], promoting the exposure of polar functional groups (─COO^−^) at the material surface and thereby increasing shear strength [42, 43]. Such unique salt‐triggered adhesion enhancement highlights the potential of ZPEs in high‐concentration systems, meriting systematic future exploration.

To observe the interaction between zwitterions and TFSI^−^, the C‐O‐C signal intensity was normalized in the FTIR spectrum, since SPEs contain identical molar ratios of PEG segments (Figure 2g). MPAE and MDEAE exhibit similar signals, while ZPE shows a distinct shift, indicating an interaction between the zwitterions and TFSI^−^ [28, 44]. We measured the presence of residual solvents using TGA‐IR to ensure the reliability of subsequent electrochemical measurements. The representative ZPE sample showed no mass loss or detectable FTIR signals below 200°C (Figure 2h). Decomposition commenced above 200 °C, evidenced by the emergence of a CO_2_ signal (∼2350 cm^−^^1^) [45]. These results confirm the solvent‐free nature of the SPEs prepared by our method. Finally, SEM imaging combined with energy dispersive spectrometer (EDS) mapping verified intimate contact between the ZPE electrolyte and LiFePO_4_ (LFP) particles in the composite cathodes (Figure 2i and Figure S7), ensuring minimal interfacial resistance for battery testing.

### 2.3 Electrochemical Characterization

**FIGURE 3:**
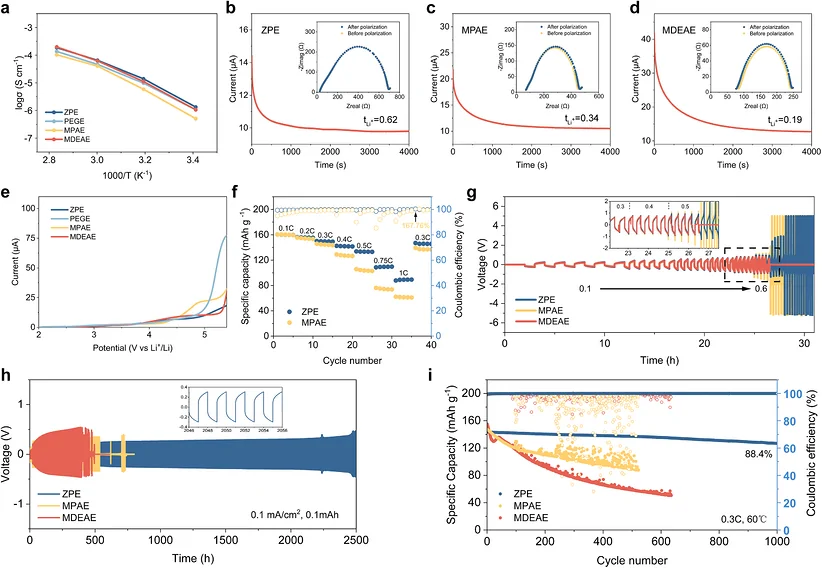
Electrochemical characterization of SPEs. (a) Ionic conductivity and the corresponding Arrhenius plots of these SPEs. (b‐d) Chronoamperometry profiles and AC impedance spectra before and after polarization (inset) for symmetric Li|SPEs|Li cell. (e) Linear sweep voltammetry (LSV) of different SPEs. (f) Rate performance of the Li|SPEs|LFP coin cell. (g) Critical current density (CCD) tests of lithium symmetric cells using these SPEs. (h) Galvanostatic cycling of lithium symmetric cells using SPEs at 0.1 mA cm^−2^. (i) Long‐term cycling performance of Li|SPEs|LFP at 0.3C.

Ionic conductivity measurements revealed that ZPE possessed the highest ionic conductivity among these SPEs, reaching 6.5 × 10^−5^ S cm^−1^ at 60°C (Figure 3a). At room temperature, ZPE achieved 2.5 × 10^−^^6^ S cm^−^^1^, surpassing MPAE (9.7 × 10^−^^7^ S cm^−^^1^) and PEGE (1.9 × 10^−^^6^ S cm^−^^1^). Furthermore, Li|SPEs|Li cells were used to determine the $t_{Li^{+}}$ of SPEs, allowing comparison of ion transport characteristics within SPEs (Figure 3b–d and Figure S8). ZPE showed the highest $t_{Li^{+}}$ (0.62) in lithium symmetric cells, significantly surpassing PEGE (0.48), MPAE (0.34), and MDEAE (0.19). The enhanced ionic conductivity and $t_{Li^{+}}$ of ZPE can be attributed to the previously observed interaction between bicine and TFSI^−^, where the positive charge of zwitterions attracts TFSI^−^, thereby facilitating the dissociation of TFSI^−^ from Li^+^ and consequently improving both the ionic conductivity and $t_{Li^{+}}$.

Electrochemical stability of the electrolytes was initially evaluated by LSV (Figure 3e). MDEAE exhibited an oxidation onset at 3.6 V, attributed to the tertiary amine oxidation. MPAE showed decomposition around 4.2 V due to ether bond degradation [46, 47]. Notably, ZPE exhibited the widest electrochemical window, extending up to 4.8 V. Interestingly, PEGE remained stable up to a similar voltage, deviating from the typical ether degradation observed at 4.2 V. Since polymer degradation begins at the domain in contact with the electrode, variations in the current signal reflect differences in this contact. It is proposed that the abundant hydrogen bonding in PUs governs the spatial distribution of PEG segments within the polymer chains, thereby contributing to the enhanced stability of PEGE. Additionally, vulnerable ether bonds are effectively shielded at the electrode interface, as the zwitterions in ZPE enhance interchain interactions and alter the local polymer conformation.

Rate capability evaluations in Li|SPEs|LFP cells (Figure 3f and Figure S9) highlighted ZPE's superior performance over MPAE. Due to recurrent short‐circuiting of MDEAE and PEGE at high current densities, testing was restricted to ZPE and MPAE systems. ZPE maintained significantly higher capacities and more stable coulombic efficiency, reflecting its faster Li^+^ transport kinetics of ZPE.

Uniform Li deposition is crucial for stable cycling in lithium metal batteries. Galvanostatic stripping and plating in Li|SPEs|Li cells revealed significant differences (Figure 3g). MDEAE experienced soft shorts at 0.3 mA/cm^2^, consistent with its poor mechanical strength [48]. MPAE exhibited rapidly increasing overpotential, exceeding instrument limits at 0.6 mA/cm^2^. In contrast, ZPE sustained stable cycling up to 0.7 mA/cm^2^. Long‐term symmetric cell cycling at 0.1 mA/cm^2^ (Figure 3h) further confirmed the exceptional stability of ZPE, manifesting stable operation for >2500 h with minimal overpotential. This performance sharply contrasts with MDEAE (short‐circuit at ∼450 h) and MPAE (short‐circuit at ∼540 h), demonstrating the effectiveness of zwitterions in regulating Li^+^ deposition/stripping and suppressing dendrite growth.

Finally, long‐term cycling was conducted using ASSLMBs (Li|SPEs|LFP) to examine the stability of SPEs (Figure 3i and Figure S10). Cells using MDEAE or MPAE exhibited rapid capacity fade, low coulombic efficiency, and early shorts, indicating unstable SEI formation due to dendrite growth and fracture. Conversely, ZPE‐based cells exhibited exceptional cycling stability, retaining 88.4% of the initial capacity after 1000 cycles. These results confirm that the zwitterionic framework not only enhances ionic transport but also stabilizes the Li interface, enabling long‐life, dendrite‐suppressing operation.

### 2.4 Salt‐Induced Phase Separation of ZPE

**FIGURE 4:**
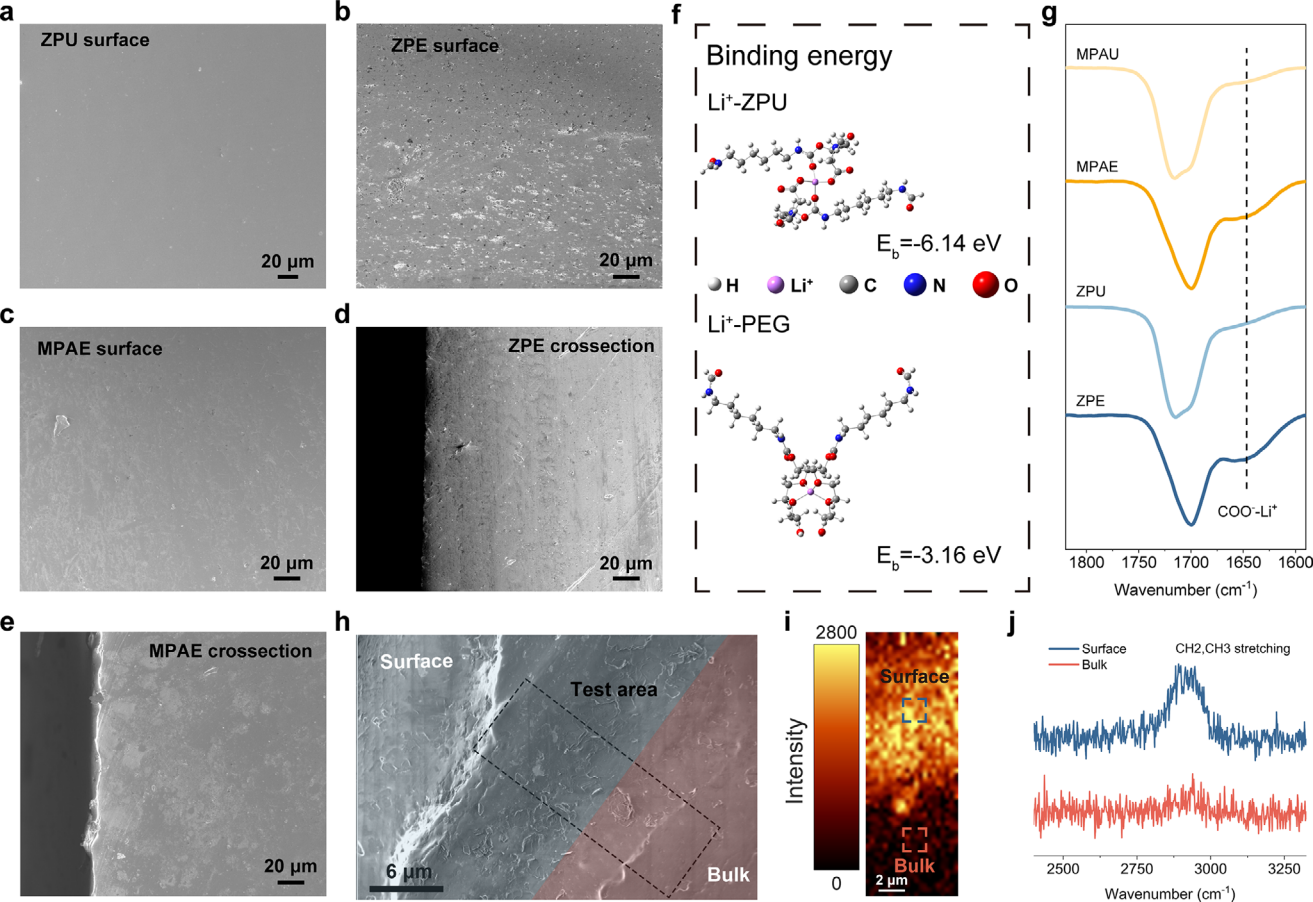
Surface phase separation characterization of SPEs. (a‐c) The surface morphology of ZPU (a), ZPE (b), and MPAE (c). (d,e) Cross‐sectional patterns of ZPE (d) and MPAE (e). f, Binding energy of Li^+^ with zwitterion segments and PEO segments in the polymer chains. (g) Changes in carboxyl groups after LiTFSI addition in FTIR spectra. (h‐j) Cross‐sectional SEM image of ZPE (h), corresponding mapping pattern through Raman spectra of test area (i), and the signals at surface area and bulk area (j).

Given the poor mechanical properties of MDEAE and its clear electrochemical inferiority to other SPEs, ZPE and MPAE were selected to elucidate the origin of their divergent lithium dendrite suppression and rate performance, despite both containing carboxyl groups. Based on the test results, although zwitterions can promote lithium salt dissociation, thereby improving ionic conductivity as well as t_Li_^+^ to some extent, this improvement was insufficient to explain such a large difference in battery cycle life. Considering the critical role of electrolyte/lithium metal interfaces in governing Li^+^ behavior, the surface characterization of the materials was initiated using SEM images. Surface observation revealed a uniform morphology for ZPU but distinct phase separation for ZPE with lithium salts (Figure 4a,b). The surface of ZPE featured irregular dark aggregates (aggregate phase) within a lighter matrix (homogeneous phase). In contrast, MPAE showed minimal aggregation and a predominantly homogeneous phase (Figure 4c).

Cross‐sectional SEM analysis further revealed depth‐dependent phase distribution differences between ZPE and MPAE (Figure 4d,e). In ZPE, the aggregates permeated approximately 20 µm beneath the surface (the sample thickness is approximately 4.5 mm), whereas MPAE aggregates were confined to superficial regions. Therefore, the difference in electrochemical properties between ZPE and MPAE can be attributed to the zwitterion‐mediated, salt‐induced phase separation.

To probe the ZPE‐Li^+^ interactions, the Li^+^ binding energies for key polymer segments (Figure 4f) were computed. The interaction between Li^+^ and the carboxyl group of the zwitterionic unit was considerably stronger (−6.14 eV) than that with PEG ether oxygens (−3.16 eV), indicating a clear preference for Li^+^ coordination to the anionic moiety. It is proposed that this pronounced binding energy difference is the likely driving force for the salt‐induced phase separation observed on the surface. Furthermore, FTIR spectroscopy revealed that, following the addition of LiTFSI, both MPAE and ZPE exhibited signals indicating the binding of carboxyl groups to lithium ions (Figure 4g) [28].

Finally, SEM‐guided Raman mapping across the ZPE cross‐section (∼20 µm depth, Figure 4h) was used to quantify phase‐specific chemical differences. Intensity mapping of the signal around 2918 cm^−^^1^ (CH stretching) [49] showed significantly stronger intensity within the region containing more aggregate phase (Figure 4i,j). This signal enhancement correlates with higher local polymer chain density and packing differences between the aggregate and homogeneous phases.

**FIGURE 5:**
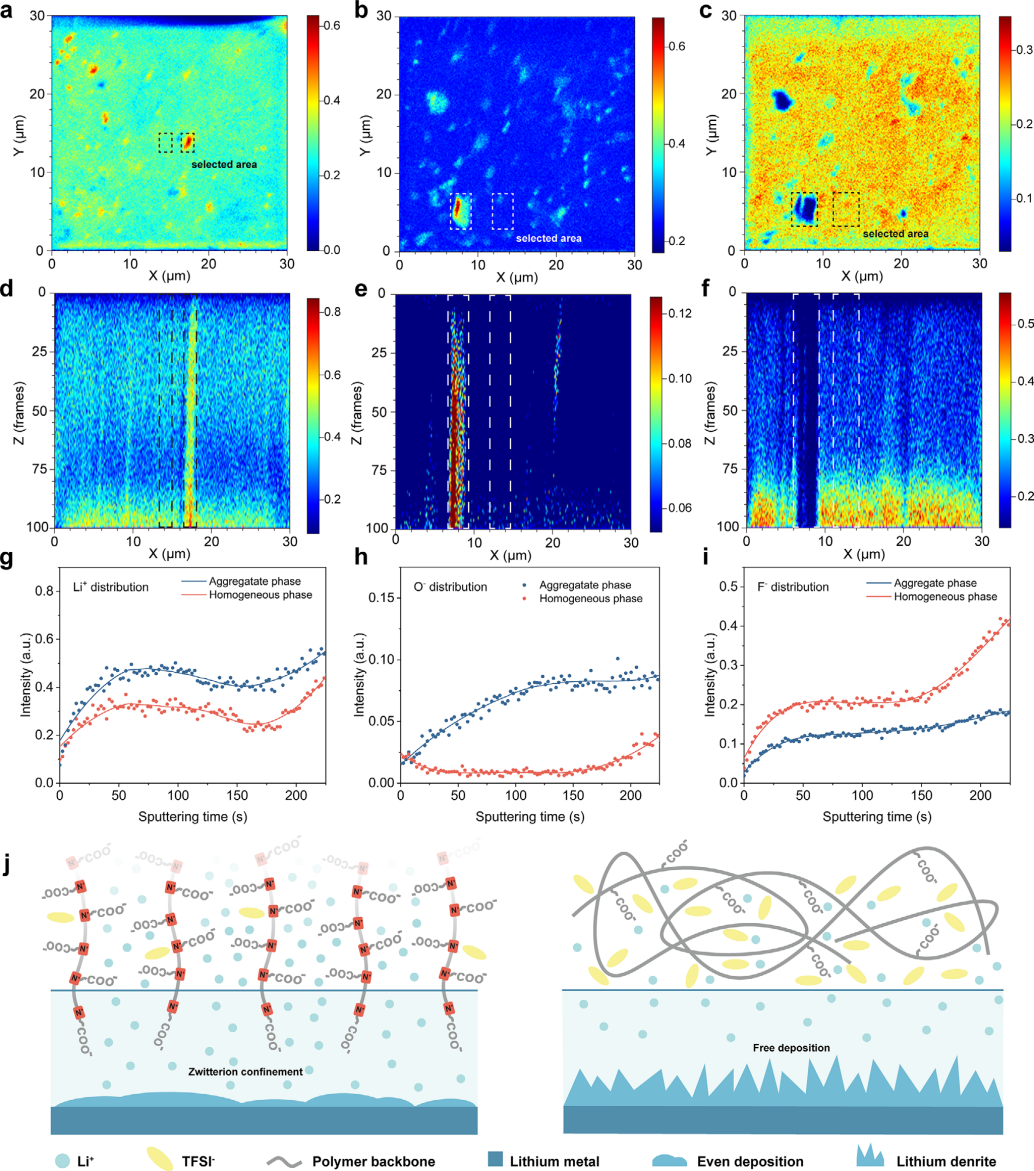
Composition analysis of different phases on the surface and mechanism of lithium ion deposition regulated by zwitterions. (a‐c) TOF‐SIMS 2D secondary ion mapping of Li^+^ (a), O^−^ (b), and F^−^ (c). d‐I, The depth profile of Li^+^ (d, g), and O^−^ (e, h), F^−^ (f, i) in selected areas. (j) The schematic diagram of the zwitterions that manipulate Li^+^ deposition.

Since Raman spectroscopy is limited by its spatial resolution (>500 nm) for phase‐specific analysis, TOF‐SIMS was employed to obtain chemical signals directly from individual aggregate domains. Mapping of anionic and cationic species across the ZPE surface (Figure S11) revealed a significantly higher Li^+^ concentration within aggregate phases compared to homogeneous phases (Figure 5a). Analysis of an intact region for anionic distribution further showed that O^−^ mapping precisely mirrored the Li^+^ pattern, with both signals concentrated in the aggregates (Figure 5b). This result directly confirms that Li^+^ binding to carboxyl groups induces the co‐aggregation of Li^+^ and O^−^ ions into shared regions, thereby confirming the computational results and FTIR spectrums.

Besides the O^−^ signal collected from the anions, the F^−^ signal from LiTFSI was also observed, revealing a striking contrast with the O^−^. The distribution of F^−^ was found to be inversely correlated with that of O^−^ and Li^+^. F^−^ was predominantly localized in homogeneous phases, in contrast to O^−^ and Li^+^ (Figure 5c). This inverse correlation confirms that the anions of zwitterions interact with Li^+^, while the cations interact with TFSI^−^, driving Li^+^ dissociation from TFSI^−^. Electrostatic repulsion and steric hindrance then exclude TFSI^−^ from the Li^+^/zwitterion‐rich aggregates, creating an environment like “ion‐enrichment pathway” within these regions. This phenomenon demonstrates the strong regulation effect of zwitterions on the ion distribution, which is directly linked to their exceptional electrochemical performance.

Two representative areas in the test region were selected as the aggregate phase and the homogeneous phase, respectively, to analyze the distribution of ions in the depth direction (Figure 5d–i). Depth profiling within selected areas confirmed the three‐dimensional ion heterogeneity, consistent with the SEM‐observed aggregate phase distribution. The signal intensity of aggregated Li^+^ is about 1.5 times greater than that of Li^+^ in the homogeneous phase, and the signal intensity of aggregated O^−^ is about eight times stronger than that of O^−^ in the homogeneous phase. The distribution of F^−^ also exhibits significant differences, with the F^−^ intensity in the homogeneous phase being approximately twice that in the aggregated phase. These differences remaining at various depths indicate that the non‐uniform spatial distribution of ions exhibits three‐dimensional connectivity, which validates the concept of “ion‐enrichment pathway”.

Although salt‐induced phase separation behavior has been observed on the surface of ZPE, leading to uneven distribution of ions between the two phases, two crucial questions remain. Why does MPA, despite containing the same anionic group, not exhibit comparable phase separation? Furthermore, why is phase separation confined to the material surface? These differences stem from the distinct intermolecular forces: electrostatic attraction between zwitterionic chains in ZPU facilitates large‐scale chain aggregation, which is necessary for phase separation. However, the repulsion between anionic chains in MPAE inhibits such clustering. Despite both zwitterionic (ZPE) and anion‐matched (MPAE) SPEs containing identical carboxyl groups, MPAE fails to develop the macroscale phase‐separated morphology observed on ZPE surfaces. The observed surface‐confined phase separation in ZPE can be explained by its preparation conditions. During fabrication, one side of the film contacted a hydrophobic environment (PTFE/LFP electrode), while the other was exposed to air. This asymmetry induces a distinct hydrophilic–hydrophobic contrast, driving polar molecules (such as zwitterions) to preferentially segregate toward the air‐facing surface. Consequently, the polymer chains adopted different orientations and structures on the upper and lower surfaces, ultimately leading to distinct surface morphologies.

In summary, the three‐dimensional ion heterogeneity driven by zwitterion‐mediated phase separation is critical for enhanced Li deposition uniformity observed in ZPE compared to MPAE. Based on these findings, a mechanistic model for the regulated Li^+^ deposition in the zwitterion system is therefore proposed, which contrasts with the unregulated deposition observed in MPAE. The stronger interaction between carboxyl groups and Li^+^ drives Li^+^ dissociation from TFSI^−^, promoting the formation of aggregated surface microdomains that function as “ion‐enrichment pathways” (Figure 5j left). Within zwitterion‐enriched aggregate phases, carboxyl groups impose spatial confinement on depositing Li^+^. This zwitterion‐mediated confinement restricts ion deposition areas, suppressing free Li^+^ diffusion and thereby promoting uniform Li^+^ deposition. In the absence of such specific interactions, cations and anions in MPAE remain uniformly distributed. Consequently, lithium ions deposit freely, leading to a higher propensity for dendrite formation (Figure 5j right). Furthermore, the formation of “ion‐enrichment pathways”‐like domains‐ shortens the diffusion path of lithium ions and enables ZPE‐based batteries to achieve better rate performance [50, 51].

### 2.5 SEI Characterization and Flexible Pouch Cell Test

Having established the role of zwitterions in regulating Li^+^ kinetics, we next examined the composition and spatial distribution of the SEI, which is equally critical for battery performance. To probe early‐stage SEI formation, the impedance evolution of Li|ZPE|LFP cells was monitored during initial cycles at 0.1C (Figure S12). The impedance stabilized by the third cycle, indicating the formation of the stable SEI. After the formation of SEI, the cell exhibited decreased polarization during charge/discharge (Figure S13), leading to an increase in capacity in early cycles.

**FIGURE 6:**
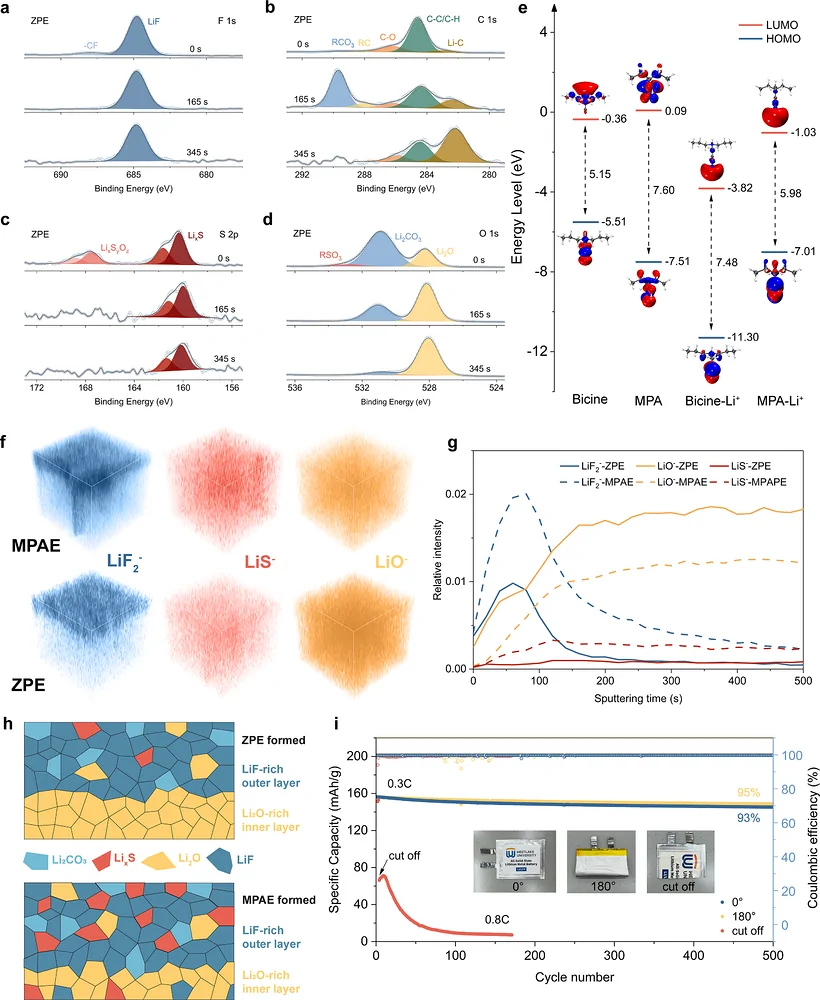
The effect of zwitterions on the SEI formation of LMBs and the performance of punch cells. (a‐d) The F 1s (a), C 1s (b), S 2p (c), O 1s (d) in‐depth XPS spectra of ZPE‐formed SEI after 3 cycles. (e) Variation of the HOMO‐LUMO energy levels of bicine, MPA, and their Li^+^‐bound structures. (f) 3D rendering of TOF‐SIMS secondary‐ion fragments sputtered from the lithium electrodes after 3 cycles in Li|MPAE|LFP cell and Li|ZPE|LFP cell. (g) Changes in representative components in SEI with increasing sputtering time. (h) Schematic of the SEI structure formed by ZPE and MPAE. (i) Cycle life of Li|ZPE|LFP punch cells under different conditions.

Therefore, the cells were disassembled after three cycles in an argon atmosphere for in‐depth XPS analysis of the Li metal surface (Figure 6a–d and Figure S14). The results revealed distinct composition differences in the SEI layers formed between the ZPE and MPAE. In the absence of a liquid electrolyte, the SEI predominantly originates from the decomposition of TFSI^−^ from LiTFSI and the reduction of polymer, leading to the formation of LiF, Li_2_O, Li_x_S, and Li_x_S_y_O_z_ compounds [52, 53, 54]. Initially, a weak C–F signal was detected in the F 1s spectrum and disappeared after 5 s of etching (Figure S15). This signal is attributed to residual LiTFSI adsorbed on the lithium metal surface. In addition to LiF, spectral features corresponding to Li_x_S_y_O_z_ and Li_x_S were identified in the outer SEI layer. The Li_x_S_y_O_z_ signal disappeared after 165 s of etching, and the Li_X_S signal also continued to weaken. Simultaneously, the intensity of the Li_2_O signal in the O1s spectrum increased with depth, while the signal of Li_2_CO_3_ continuously attenuated, indicating that the interior of the SEI was primarily composed of Li_2_O. The SEI compositions formed from ZPE and MPAE contained similar chemical species, stemming from the decomposition of TFSI^−^. The primary distinction lies in the relative abundance and spatial distribution of these components within the SEI.

For SPEs, the polymer decomposition also occurs on the lithium metal surface, contributing to the formation of the SEI. Due to the strong binding interaction between carboxyl groups and lithium ions, we calculated the HOMO‐LUMO energy level structures of bicine and MPA prior to Li^+^ binding, as well as the energy level structures after binding with a Li^+^ (Bicine‐Li^+^ and MPA‐Li^+^) (Figure 6e and Figure S16). When binding Li^+^ through carboxyl groups, bicine and MPA display distinct behaviors. Before binding with Li^+^, bicine exhibits a higher HOMO energy level of −5.51 eV compared to MPA's −7.51 eV and a lower LUMO energy level of −0.36 eV relative to MPA's +0.09 eV. This smaller band gap of 5.15 eV compared to 7.60 eV makes bicine inherently more reactive in its pristine state. After Li^+^ coordination, Bicine‐Li^+^ achieves the most negative LUMO at −3.82 eV, promoting reductive decomposition for effective SEI formation at the anode. Additionally, bicine‐Li^+^ exhibits the lowest HOMO energy level (−11.3 eV), indicating that binding with lithium ions enhances the oxidative stability of bicine. This contributes to improved stability on the cathode side of the battery.

Furthermore, TOF‐SIMS was used to analyze the structure of the SEI. This technique provided complementary data on the distribution of anion‐derived fragments within the SEI layers. Specifically, signals corresponding to LiF and Li_x_S showed higher intensities in the MPAE‐formed SEI compared to that formed with ZPE, whereas the Li_2_O signal exhibits stronger intensity in the ZPE‐formed SEI (Figure 6f,g). It is hypothesized that the binding of bicine to Li^+^ induces a reduction process on the lithium metal surface, producing additional Li_2_O. Depth‐profiling TOF‐SIMS analysis further revealed distinct distribution patterns of these fragments. LiF and Li_x_S were primarily located near the SEI surface, while Li_2_O was concentrated in deeper regions. Consequently, the ZPE‐derived SEI exhibited a thinner surface layer composed of LiF and Li_x_S, and a more pronounced enrichment of Li_2_O in the inner layer. This structure differed significantly from that of the MPAE‐formed SEI in both composition and distribution. Therefore, it is clear that ZPE and MPAE promoted the formation of two distinct SEI architectures (Figure 6h). The ZPE‐formed SEI featured a denser and more homogeneous inner layer enriched with Li_2_O, along with an outer layer composed primarily of LiF. Li_2_O is known to facilitate homogeneous lithium plating owing to its high Li^+^ conductivity [55, 56]. By promoting a gradient SEI structure comprising a Li_2_O‐dominated inner layer and a LiF‐dominated outer layer, ZPE effectively suppresses the growth of lithium dendrites.

Collectively, these results demonstrate that surface phase separation in ZPE governs the inhomogeneous distribution of lithium salts and facilitates the formation of Li_2_O on lithium metal, critically impacting both Li^+^ deposition uniformity and SEI properties. Through investigation of surface morphology, ion distribution, SEI structure, and formation mechanism, we believe that the combination of surface ion flux regulation with SEI engineering is the key to the significant extension of lithium metal battery cycle life by ZPE. This improvement was achieved through a combination of mechanisms, not solely through the ability of zwitterions to promote lithium salt dissociation.

Finally, leveraging ZPE's excellent adhesion and flexibility, its applicability was further validated in flexible Li|ZPE|LFP pouch cells. These cells maintained stable open‐circuit voltage under flat (0°), 90°, and 180° bending (Figures S17 and S18). Remarkably, galvanostatic cycling without external pressure achieved high‐capacity retentions of 93% (flat) and 95% (180° bent) over 500 cycles (Figure 6i), thus verifying robust dendrite suppression on curved electrodes.

After being cut in half, the battery continued operating at the same current (0.8C for the decreased electrode area). This sustained operation underscores the robust interfacial contact maintained by ZPE even after mechanical damage. Although a gradual capacity fade occurred, likely due to ambient moisture exposure, the absence of fire or smoke unequivocally demonstrates the intrinsic safety of ZPE credentials. ZPE demonstrated competitive performance under solvent‐free conditions, matching results reported in the literature (Table S2).

## 3 Conclusion

In summary, four distinct PUs were synthesized, and corresponding SPEs were investigated to elucidate the functions of zwitterions in ASSLMBs. Systematic comparisons revealed ZPE can effectively improve the mechanical and electrochemical properties of SPEs and exhibit unique behaviors. Crucially, these behaviors are primarily associated with the distinctive “anti‐polyelectrolyte effect” of zwitterions, which also explains the divergent behaviors observed between ZPE and structurally analogous MPAE. The zwitterion‐mediated, salt‐induced phase separation on the ZPE surface underpins its outstanding electrochemical performance. This phenomenon originates from strong zwitterion‐Li^+^ binding and zwitterion‐zwitterion attraction. These interactions drive phase separation, which in turn promotes Li^+^‐TFSI^−^ dissociation, yielding a Li^+^‐enriched aggregate phase and a TFSI^−^‐rich homogeneous phase. Within the aggregate phase, the assembly of zwitterions forms “ion‐enrichment pathway”‐like domains. These domains may impose spatial confinement on the lithium metal surface and regulate Li^+^ flux, mediating uniform deposition. Furthermore, Li^+^ coordination significantly lowers the LUMO energy of bicine and enhances its reduction tendency. This facilitates the formation of a stable, Li_2_O‐rich inner SEI layer on the lithium metal surface that accelerates Li^+^ transport and effectively suppresses dendrite growth as well. Therefore, we suggest that the ion flux regulation on the surface of ZPE, and the SEI architecture engineering collectively promote uniform Li^+^ deposition. Our work provides a better understanding of the working principle of zwitterionic polyelectrolytes in ASSLMBs and sheds light on the application of polymer ASSLMBs for safe and advanced energy storage systems.

## Conflicts of Interest

The authors declare no conflicts of interest.

## Supporting information




**Supporting File**: advs76224‐sup‐0001‐SuppMat.docx.

## Data Availability

The data that support the findings of this study are available in the supplementary material of this article.
