# Supplementary material for: Revealing the Ion Regulation Effect of Zwitterionic All‐Solid‐State Electrolytes in Lithium Metal Batteries
Source: Adv Sci (Weinh). 2026 Jun 22;13(52):e76224. doi: 10.1002/advs.76224 (PMC13336380; doi:10.1002/advs.76224)
Supplement: Supplementary file 1 [file ADVS-13-e76224-s001.docx]

Revealing the ion regulation effect of zwitterionic all-solid-state electrolytes in lithium metal batteries

*Wentao Xie^a, b^, Yu Wu^b^, Yierfan^b^, Yuling Yang^c^, Dongdong Gao^b^, liangbo Xu^b^, Luoman Qin^d^, Xingyu Lu^e^,* *Yinjuan chen^e^,* *Changle Mu^f^, Danyu Gu^e^, Chunlei Wei^g, h^, Yi He^g, h^, Gang Cheng^b, i^****^*^***

Add the affiliation for every authors

^a^ College of Materials Science and Engineering, Zhejiang University, Hangzhou, 310058, China

^b^ School of Engineering, Westlake University, Hangzhou, 310024, China

^c^ School of Science, Westlake University, Hangzhou, 310024, China

^d^ School of Life Sciences, Westlake University, Hangzhou, 310024, China

^e^ Zhejiang Key Laboratory of Precise Synthesis of Functional Molecules, Instrumentation and Service Center for Molecular Sciences, Westlake University, Hangzhou, 310030, China

^f^ Instrumentation and Service Center for Physical Sciences, Westlake University, Hangzhou 310024, China

^g^ College of Chemical and Biological Engineering, Zhejiang University, Hangzhou, 310058, China

^h^ Institute of Zhejiang University-Quzhou, Quzhou, 324000, China

^i^ Center for Biobased Materials, Muyuan Laboratory, Zhengzhou, 450016, China

Keywords: all-solid-state, polymer, lithium battery, polyelectrolyte

**Corresponding author: Gang Cheng,** [chenggang@westlake.edu.cn](mailto:chenggang@westlake.edu.cn)

**Material synthesis**

**Method**

**(1) Material preparation**

**Zwitterionic polyurethane (ZPU) synthesis:** Zwitterionic polyurethane (ZPU) was synthesized as follows. N,N-bis(2-hydroxyethyl)glycine (Bicine, TCI, ≥99%, 0.2 mol), poly(ethylene glycol) (PEG, Mw = 1000, Sigma-Aldrich, 0.6 mol), and 3-allyloxy-1,2-propanediol (AOPE, TCI, ≥99%, 0.2 mol). were introduced into a three-necked flask. Prior to use, PEG was preheated in the vacuum oven at 80 ℃ to remove moisture. The three-necked flask was alternately evacuated and purged with nitrogen three times to eliminate residual water and ensure an inert atmosphere. After this step, a continuous nitrogen flow was maintained to preserve positive pressure and prevent the ingress of moisture and oxygen from the air.

The monomer mixture was stirred mechanically, and then hexamethylene diisocyanate (HDI, Macklin, ≥99%) was added dropwise at 80 °C using a syringe. The mouth of the flask for adding raw materials was sealed with a rubber plug to minimize exposure to air during addition. After two hours of the reaction, dibutyltin dilaurate (DBTDL, Sigma-Aldrich, ≥95%) was added as a catalyst, and polymerization proceeded at 80°C for 24 hours. Anhydrous N,N-Dimethylformamide (DMF) was employed as the solvent to control the viscosity during the reaction. The reaction was terminated by adding 5 ml of anhydrous ethanol dropwise at 60 °C.

The resulting polymer was purified by precipitation with ether and chloroform three times to remove DMF, unreacted monomers, and low-molecular-weight residues.

**Zwitterionic polymeric electrolyte (ZPE) preparation:** 2 g ZPU, 1.08 g lithium bis (trifluoromethane)sulfonimide (LiTFSI) (Macklin, ≥99.9%) were dissolved in methanol to obtain a homogeneous ZPE precursor solution with a total concentration of 2.18 g/ml in a centrifuge tube. 2,2-Dimethoxy-2-phenylacetophenone (DMPA, Adamas-beta, ≥99%), and 2,2′-(Ethylenedioxy) diethanethiol (EDT, Crosslinker, Macklin, ≥95%) were the employed as the photoinitiator and crosslinker, respectively. Appropriate amounts of DMPA and EDT were added according to the following formulas (1) and (2), and the mixture was degassed by high-speed centrifugation to remove air bubbles. The well-mixed ZPE solution was cast onto a Polytetrafluoroethylene (PTFE) substrate using a blade coating method. The coated films were subsequently crosslinked by ultraviolet (UV) irradiation for 5 minutes. The resulting crosslinked ZPE films were then dried in a vacuum oven at 120 °C for more than 12 hours to remove residual solvent. The prepared ZPE were stored in a glove box (H_2_O≤0.01 ppm, O_2_≤0.01 ppm) for subsequent characterizations.

$\text{m}_{\text{Initiator}}\text{=}\frac{\text{M}_{\text{ZPU}}}{\text{M}_{\text{Bicine}}\text{+}\text{M}_{\text{PEG}}\text{+}\text{M}_{\text{AOPE}}}\text{×}\text{N}_{\text{AOPE}}\text{÷10×256.31}$ (1)

$\text{m}_{\text{Crosslinker}}\text{=}\frac{\text{M}_{\text{ZPU}}}{\text{M}_{\text{Bicine}}\text{+}\text{M}_{\text{PEG}}\text{+}\text{M}_{\text{AOPE}}}\text{×}\text{N}_{\text{AOPE}}\text{÷2×183.2}$ (2)

Where $\text{m}_{\text{Initiator}}$, and $\text{m}_{\text{Crosslinker}}$ represent the respective masses of DMPA and EDT added to the ZPE solution. $\text{M}_{\text{ZPU}}$ is the mass of ZPU. $\text{M}_{\text{Bicine}}$, $\text{M}_{\text{PEG}}$,and $\text{M}_{\text{AOPE}}$ correspond to mass of Bicine, PEG, and AOPE used during ZPU synthesis. $\text{N}_{\text{AOPE}}$ is the amount of molar number of AOPE during the synthesis process. The molar masses of DMPA and EDT are 256.31 g/mol and 183.2 g/mol, respectively.

**MPAU, MDEAU, and PEGU synthesis:** The synthesis procedures for MPAU and MDEAU are identical to those for ZPU, except that Bicine is replaced with 2,2-Bis(hydroxymethyl)propionic acid (MPA, Sigma-Aldrich, 98% and *N*-methyldiethanolamine (MDEA, Sigma-Aldrich, ≥99%), respectively. The synthesis procedure for PEGU was similar to that for ZPU, except that during the addition of reagents, the amount of PEG was adjusted to 0.8 mol, while the amount of AOPE remains at 0.2 mol.

**MPAE, MDEAE, and PEGE preparation:** Similarly, the subsequent procedures for preparing MPAE, MDEAE and PEGE were the same as those for preparing ZPE, except that the polymer in the precursor solution was changed.

**Cathode and composite electrolyte preparation**

LiFePO_4_ (LFP) cathode was prepared by mixing active material, Super P, and PVDF in a weight ratio of 8:1:1 using N-Methylpyrrolidone (NMP) as the solvent to form a homogeneous slurry. Then the slurry was cast on aluminium foil and dried under vacuum at 120 °C for 12 h to completely remove the NMP solvent. The resulting cathode exhibited a mass loading of 2.5 mg/cm^2^.

The LFP composite electrode was prepared by casting the precursor solution of the SPEs directly onto the surface of the LFP cathode. After ensuring full wetting of the electrode surface, the film was UV-crosslinked in situ. The obtained composite electrode was subsequently dried under vaccum at 120 ℃ for 12 h to eliminate any residual solvent. Finally, the fully dried composite electrode was transferred into an argon-filled glove box for further characterization.

**Materials characterizations:**

Attenuated Total Reflectance-Fourier Transform Infrared Spectroscopy (ATR-FTIR) spectra were collected using a Thermo Scientific Nicolet iS50. Dynamic thermomechanical analysis (DMA) was performed on a TA-Waters Discovery DMA 850 instrument at a heating rate of 10 ℃ min^-1^. Tensile and lap-shear tests were carried out on a Universal Testing Machine (UTM). For tensile testing, the samples were cut into dumbbell-shaped specimens with a central dimension of 8×5 mm. For lap-shear and 180° peel tests, two pieces of aluminum foil were glued together using the prepared material, with an adhesion area of ~10×20 mm. Thermogravimetric analysis/infrared spectroscopy (TGA-IR) measurement was conducted using a Mettler Toledo TGA/DSC 3+ and a Thermo Scientific Nicolet iS50 at a heating rate of 10 °C min^−1^.

The sample of composite electrode scanning electron microscopy (SEM) and energy-dispersive spectroscopy (EDS) were taken with a field emission environment scanning electron microscopy (FE-SEM, Thermo Scientific Quattro S) at 3 kV for observing the cross-sectional image of composite electrode. Surface phase separation was characterized by Raman spectroscopy using a by Zeiss Gemini460 at 2 kV. Cross-sectional samples were prepared by vertically fracturing the film with a blade. X-ray diffraction (XRD) patterns were obtained on a Bruker D8 Advance. X-ray Photoelectron Spectroscopy (XPS) measurements were conducted on a Thermo Scientific Nexsa G2 system. Time-of-flight secondary ion mass spectrometry (TOF-SIMS) were analyzed by Thermo Scientific Helios 5 UX and ULVAC-PHI NanoTOF 3 instrument. After three charge-discharge cycles of Li|SPEs|LFP coin cells at 0.1 C, the cells were disassembled inside an argon-filled glovebox (O_2_ and H_2_O <0.1 ppm). The recovered samples were transferred via a sealed chamber for XPS and TOF-SIMS measurements to analyze the composition and structure of the solid electrolyte interphase) (SEI).

**Electrochemical measurements:**

The ionic conductivities of solid-state polymer electrolytes (SPEs) at various temperatures (20–80  °C) were determined by electrochemical impedance spectroscopy on electrochemical workstation (Garmy Interface 1010E) over a frequency range of 2.0  MHz to 0.1  Hz. The measurements were performed on stainless steel (SS) symmetric coin cell SS|SPEs|SS with SS and SPE diameter of 2 mm and 19 mm, respectively. The ionic conductivities were calculated as follows:

$\sigma=\frac{L}{RS}$ (3)

where L is the thickness of the SPEs, R is the resistance of the electrolyte, and S is the area of SS electrodes. The lithium-ion transference number (t_+_) was determine at 60 ℃ using a Li|Spe|Li symmetric cells with a lithium electrode diameter of 15.8 mm. The t_+_ was calculated as follows:

$t_{+}=\frac{I_{s}(\Delta V-I_{0}R_{0})}{I_{0}(\Delta V-I_{s}R_{s})}$ (4)

where ∆V is the applied voltage polarization applied (10 mV), I_s_ and R_s_ are the steady-state current and resistance, respectively. I_0_ and R_0_ are the initial current and resistance, respectively.

Linear sweep voltammetry (LSV) was conducted on Li|SPEs|SS coin cells with a scan rate of 1 mV s^-1^ on electrochemical workstation. The charge and discharge C-rate of the batteries were calculated based on 1C = 170 mAh/g. Critical current density (CCD) tests were carried out by assembling Li|SPEs|Li coin cells and cycled at in creasing current density from 0.1 mA cm^-2^ to 0.7 mA cm^-2^ with a fixed capacity of 0.1 mAh. For Galvanostatic cycling of Li|SPEs|Li cell, the current density was set to 0.05 mA cm^-2^ at beginning of 5 cycles and then increased to 0.1 mA cm^-2^ for subsquent cycles, with a fixed capacity of 0.1 mAh at 60 ℃. The cycling performance of Li|SPEs|LFP cells was evaluated via galvanostatic charge-discharge test in the voltage range of 2.5–4 V for coin cells and 2.5-3.8 V for pouch cell at 60 ℃.

All the coin cells used in this work were 2032-type, and the thickness of lithium foil for pouch cell is 100 μm.

**Computational details**

**(1) Binding energy:**

Quantum chemical calculationa were carried out using density functional theory (DFT) as implemented in GAUSSIAN 16 package^1^. Geometry optimization and frequency analyses were performed using the B3LYP hybrid functional^2^ with GD3BJ dispersion correlation at 6-31G(d, p) basis sets. The bonding energy of Li^+^-Bicine and Li^+^-MPA are calculated as follows:

E_b_ = E(Li^+^-Bicine) - E(Li^+^) - E(Bicine) (5)

E_b_ = E(Li^+^-MPA) - E(Li^+^) - E(MPA). (6)

**(2) HOMO-LUMO energy level**

Quantum chemical studies are performed using density functional theory (DFT) implemented in GAUSSIAN 16 package^1^. Geometry optimization and frequency analysis are calculated at B3LYP hybrid functional^2^ with GD3BJ dispersion correlation at 6-311+G(d, p) basis sets. Molecular orbitals (MOs) are performed by Multiwfn 3.8^3^ and VMD v 1.9.3^4^ molecular visualization software.

**Figure S1|** The optical images of the ZPE solution crosslinked by UV irradiation.

**Figure S2|** FTIR spectrum of PUs and SPEs

**Figure S3|** XPS signals of ZPU. N 1s (a), O 1s (b), S 2p (c).

**Figure S4|** Puncture of ZPU film with 1 ml pipette tip

**Figure S5| a.** Storage modulus of ZPU at room temperature and 60 ℃. **b.** Storage modulus of ZPU varied with increasing temperature.

While solid-state electrolytes mitigate safety concerns associated with liquid electrolyte leakage, their mechanical performance can be strongly influenced by temperature variations. Dynamic mechanical analysis (DMA) was conducted to evaluate the temperature-dependent mechanical properties of ZPU. The storage modulus of ZPU was stable below 150 ℃, which is higher than the shutdown temperature of commercial separator (~135 ℃).

**Figure S6| a.** Illustration of the shear strength setting up where the material was placed between two aluminum sheets and the glued area was 2cm x 1cm in size. **b.** 180° peeling test of ZPU and ZPE. **c.** Demonstration of ZPE adhesion strength—two aluminum sheets bonded with ZPE were used to lift a 5.5 L water bottle (~5.5 kg), confirming the outstanding adhesive performance of ZPE.

**Figure S7|** EDS mapping of LPF-ZPE composite

**Figure S8|** Chronoamperometry profiles and AC impedance spectra before and after polarization (inset) for symmetric Li|PEGE|Li cell.

**Figure S9|** Galvanostatic charge–discharge voltage profiles of the cell using ZPE at different C rate.

When Li|ZPE|LFP coin cells are charged and discharged at rates ranging from 0.1C to 1C, their specific capacities are 159.6, 156.2, 150.2, 142.6, 133.8, 109.7, and 89.3 mAh/g, respectively.

**Figure S10|** The variation of capacity with increasing cycle numbers.

The charge-discharge curves of ZPE during the cycle process show excellent complianc, proving that the structure of the cell can remain stable during the cycle process.

**Figure S11|** The focused ion beam (FIB) image of ZPU surface. **a, b,** The selected areas for cation signals (a) and anion signals (b) collection.

**Figure S12|** The Nyquist plots of Li|ZPE|LFP coin cells varies with the number of cycles.

**Figure S13|** The charge/discharge voltage profile of Li|ZPE|LFP coin cells during the SEI formation process.

**Figure S14|** The F 1s **(a)**, C 1s **(b)**, S 2p **(c)**, O 1s **(d)** in-depth XPS spectra of MPAE-formed SEI after 3 cycles.

**Figure S15|** The F 1s spectra of SEI formed by ZPE and MPAE after etching 5s.

**Figure S16|** The structures for calculating HOMO-LUMO energy levels.

**Figure S17**| Open circuit voltage changes at different states of punch cells, pristine (a), 90° bending (b), 180° bending (c), and the cell was cut in half (d)

**Figure S18|** The cross section of LMB after cutting off.

**Supplementary Table S1.** Mechanical properties of materials at room temperature

|  | **MDEAU** | **MPAU** | **ZPU** |
| --- | --- | --- | --- |
| Elastic modulus | 0.3 MPa | 1.3 MPa | 2.0 MPa |
| Elongation at break | 464 % | 673 % | 579 % |
| Toughness | 1.16 MJ/m^3^ | 11.83 MJ/m^3^ | 12.34 MJ/m^3^ |

**Supplementary Table S2. The Comparison of the solid-state electrolyte reported in literature**

**Reference**

1	Frisch, M., Trucks, G., Schlegel, H., Scuseria, G., Robb, M., Cheeseman, J., Scalmani, G., Barone, V., Petersson, G., and Nakatsuji, H. (2016). Gaussian 16, Revision C. 01 Normal name order. Gaussian.

2	Stephens, P.J., Devlin, F.J., Chabalowski, C.F., and Frisch, M.J. (1994). Ab initio calculation of vibrational absorption and circular dichroism spectra using density functional force fields. *The Journal of physical chemistry* *98*, 11623-11627.

3	Lu, T., and Chen, F. (2012). Multiwfn: a multifunctional wavefunction analyzer. *Journal of computational chemistry* 33, 580-592.

4	Humphrey, W., Dalke, A., and Schulten, K. (1996). VMD: visual molecular dynamics. *Journal of molecular graphics* 14, 33-38.

5	Chen, Q., Tang, W., Gu, T. & Wang, D. Y. Bifunctional Polyrotaxane‐Based Electrolytes Enable Long‐Cycling and Fire‐Safe All‐Solid‐State Lithium Metal Batteries. *Advanced Functional Materials*, e26101 (2025).

6	Chen, J. *et al.* Multiple Dynamic Bonds‐Driven Integrated Cathode/Polymer Electrolyte for Stable All‐Solid‐State Lithium Metal Batteries. *Angewandte Chemie International Edition* **62**, e202307255 (2023).

7	Mu, J. *et al.* Constructing Gradient Soft‐Rigid Structure for Directed and Fast Li‐Ion Transfer Channels in Composite Solid Electrolytes. *Advanced Functional Materials,* e19281 (2025).

8	Kong, Z. *et al.* Balanced Anion-Cation-EO Interaction Enables Ultrahigh Lithium-Ion Transport in 4.5 V-Class PEO-Based All-Solid-State Lithium Batteries. *Advanced Materials* **38**, e14236 (2026).

9	Wang, L. et al. Deep Eutectic Interaction Induced Lithium Salt Dissociation for Inorganic‐Rich Solid Electrolyte Interphase in All‐Solid‐State Batteries. *Advanced Functional Materials* 36, e16694 (2025).

10	Fan, Y. *et al.* Surface-Confined Disordered Hydrogen Bonds Enable Efficient Lithium Transport in All-Solid-State PEO-Based Lithium Battery. *Angewandte Chemie International Edition* **64**, e202421777 (2025).

11	Yue, K. *et al.* Low-LUMO Orotic Acid Enables Li(3)N-Embeded Solid Electrolyte Interphase for Stable All-Solid-State Lithium Metal Batteries. *Small* **21**, e07263 (2025).

12	Tang, D. et al. Ion‐Selective Pumping and Entropy‐Driven Lithium Transport in Composite Electrolytes via Dynamic Competitive Coordination for All‐Solid‐State Batteries. *Advanced Functional Materials*, e17250 (2025).

13	Hou, J. *et al.* Multiscale Engineered Bionic Solid-State Electrolytes Breaking the Stiffness-Damping Trade-Off. *Angewandte Chemie International Edition* **64**, e202421427 (2025).

14	Li, M., Yang, W., Sun, L., Li, Y. & Chen, X. Al-doped garnet nanofiber-reinforced cathode-supported composite solid electrolyte membranes for advanced all solid-state lithium batteries. *Journal of Energy Storage* **118**, 116247 (2025).

15	Yan, S. S., Wang, Z., Liu, F. X., Zhou, H. Y. & Liu, K. Aromatic Donor-Acceptor Charge-Transfer Interactions Reinforced Supramolecular Polymer Electrolyte for Solid-State Lithium Batteries. *Advanced Functional Materials* ,2303739 (2023).
